# Associations between attributes of live poultry trade and HPAI H5N1 outbreaks: a descriptive and network analysis study in northern Vietnam

**DOI:** 10.1186/1746-6148-6-10

**Published:** 2010-02-22

**Authors:** Ricardo J Soares Magalhães, Angel Ortiz-Pelaez, Kim Lan Lai Thi, Quoc Hoang Dinh, Joachim Otte, Dirk U Pfeiffer

**Affiliations:** 1 Royal Veterinary College, Veterinary Epidemiology & Public Health Group, Dpt. Veterinary Clinical Sciences, Hawkshead Lane, North Mymms, Hatfield, Herts, AL9 7TA, UK; 2 School of Population Health, University of Queensland, Public Health Building, Herston Road, Herston QLD 4006, Australia; 3 Department of Animal Health, 15/78 Giai Phong Street, Phuong Mai Ward, Dong Da District, Ha Noi, Vietnam; 4 Food and Agriculture Organisation of the United Nations, Pro-Poor Livestock Initiative, Vialle delle Terme di Caracalla, 00153 Rome, Italy

## Abstract

**Background:**

The structure of contact between individuals plays an important role in the incursion and spread of contagious diseases in both human and animal populations. In the case of avian influenza, the movement of live birds is a well known risk factor for the geographic dissemination of the virus among poultry flocks. Live bird markets (LBM's) contribute to the epidemiology of avian influenza due to their demographic characteristics and the presence of HPAI H5N1 virus lineages. The relationship between poultry producers and live poultry traders (LPT's) that operate in LBM's has not been adequately documented in HPAI H5N1-affected SE Asian countries. The aims of this study were to document and study the flow of live poultry in a poultry trade network in northern Vietnam, and explore its potential role in the risk for HPAI H5N1 during 2003 to 2006.

**Results:**

Our results indicate that LPT's trading for less than a year and operating at retail markets are more likely to source poultry from flocks located in communes with a past history of HPAI H5N1 outbreaks during 2003 to 2006 than LPT's trading longer than a year and operating at wholesale markets. The results of the network analysis indicate that LPT's tend to link communes of similar infection status.

**Conclusions:**

Our study provides evidence which can be used for informing policies aimed at encouraging more biosecure practices of LPT's operating at authorised LBM's. The results suggest that LPT's play a role in HPAI H5N1 transmission and may contribute to perpetuating HPAI H5N1 virus circulation amongst certain groups of communes. The impact of current disease prevention and control interventions could be enhanced by disseminating information about outbreak risk and the implementation of a formal data recording scheme at LBM's for all incoming and outgoing LPT's.

## Background

The recurrence of poultry outbreaks of highly pathogenic avian influenza of the H5N1 subtype (HPAI H5N1) in some parts of the world, and the occasional spillover of infection to humans, is a significant global health concern. Poultry rearing is an important enterprise in countries across the greater Mekong region. In Vietnam, poultry rearing is closely linked with people's livelihoods and traditional culture [1,2].

Five epidemic waves of HPAI H5N1 occurred in Vietnam between late 2003 and mid-2008, causing the second highest human case incidence and case-fatality rates in the world. The proximity of poultry flocks to water courses and paddy fields, and keeping of other poultry species all have an important role in sustaining and perpetuating infection [3-7]. Poultry outbreaks are primarily reported in the Red and Mekong river deltas, and the majority of outbreaks are recorded in the predominant small-holder chicken and duck flocks. Agro-ecological factors related to poultry husbandry, trade and social-cultural customs are suggested to be associated with the maintenance of the HPAI H5N1 infection cycle in Vietnam [3,6-8]. The daily outbreak incidence during the first two epidemic waves (2003-2004 and 2004-2005) peaked around the annual "Tet" holiday festivities in February, when poultry movement is increased [6,9]. However, the temporal distribution of H5N1 outbreaks has changed since the introduction of vaccination in 2005, and since mid-2008 reported outbreaks have not shown a regular pattern [10].

In both human and animal populations, the structure of contact between individuals contributes to the incursion and spread of contagious diseases [11-13]. In the case of avian influenza, the movement of live birds is a risk factor for the dissemination of the virus to poultry flocks [14]. In particular, live bird markets have long been considered to be an important link in the pathways that lead to the emergence and reintroduction of infection. These markets facilitate the congregation of large populations of animals- that have originated from a diversity of sources in a fairly large geographical area -- in relatively small spaces [15-17]. This becomes particularly noteworthy as avian influenza surveillance studies in the United States and in Southeast (SE) Asia have provided evidence of presence of virus lineages in live bird markets [18-23]. Similarly, a virological survey in ten live bird markets in Ha Noi has shown that the HPAI H5N1 virus was already circulating in healthy geese as early as 2001 [18]. Also, evidence suggests that live bird markets can be suitable environments for potential virus re-assortment and transmission [18,24]. The viruses found in 2005 in Ha Noi's live bird markets have been reported to be genetically related to virus isolated in Hong Kong in 1997, but are genetically distinct from those isolated in northern Vietnam in early 2004 [18,25,26]. This supports the hypothesis that separate virus introductions via trade of live poultry might be responsible for different outbreak periods. This is further supported by a recent molecular study of HPAI H5N1 viruses that suggested that outbreaks in the north of Vietnam are likely to be attributable to multiple introductions of virus primarily through transboundary trade with southern China [27].

Factors such as (1) culturally-driven seasonal patterns of poultry demand and (2) the close inter-linkage of poultry production with other seasonal agricultural activities (3) and disease control interventions are expected to influence the production levels of different species and therefore their marketing patterns [1,2]. In relation to disease control, policies applied during the outbreak waves included movement restrictions, restrictions to breeding of certain poultry, and market bans that were fundamentally similar to the ones applied in Hong Kong LBM's after 1997 [6,28]. The intended effect of restrictions to live poultry trade was to reduce the exposure of humans at markets, market contamination and opportunities for virus recombination [24,29,30].

To date, the relationship between small-holder poultry production and trade, and in particular, between small-scale poultry holders, poultry traders and live bird markets has been insufficiently studied and documented in HPAI H5N1-affected SE Asian countries. This information is difficult to obtain but is essential for understanding outbreak recurrence associated with poultry trade. The available information for Vietnam does not provide substantive evidence on the association between production and trade in smallholder poultry and its role on outbreak occurrence. The objective of the present study is to better understand the flow of live poultry, as investigated in a poultry trade network of northern Vietnam, and explore its potential role in the risk for HPAI H5N1 introduction and spread and the resulting implications for disease control policies.

## Results

### Relationship between trade attributes of LPT's

The characteristics of LPT's are presented in Table 1. The LPT's that sell and buy poultry on the same day often leave the LBM and distribute poultry to several destinations other than slaughter; this trade type is performed by individuals more experienced in poultry trade. The number of communes visited by LPT's was not large (maximum: 4) nor was the number of contact flocks in them (maximum: 10). The communes included in the current study have significantly larger numbers of flocks and smaller commune areas when compared to other areas in the country (P < 0.001). The distances from commune to LBM were generally quite short (median = 8 km, range: 1-38 km), except for Ha Vi and Bac Tan Long markets. Our results also suggest that more experienced LPT's tend to trade in retail markets (χ^2 ^for trend = 12.20; p = 0.007) and that LPT's operating at wholesale markets tend to contact larger flocks (χ^2 ^for trend = 300.86, p < 0.001) and trade more poultry than those operating at retail markets (χ^2 ^for trend = 60.49; p < 0.001). The frequencies of the number of poultry traded do not differ by species of poultry (χ^2 ^for trend = 1.58; p = 0.2) but the chicken flocks contacted by LPT's tend to have large numbers of poultry than duck and Muscovy duck flocks (χ^2 ^for trend 57.56; p < 0.001).

**Table 1:** Attributes of "sell only" and "buy and sell" live poultry traders operating in all (total of 12) authorised live bird markets serving the Northern provinces of Vietnam.

Trader attribute	Direction of trade		Totals respondents
	**Sell only (%)**	**Buy and sell (%)**	

**Total number**	66 (46.48)	76 (53.52)	142

**Market type**			

*Wholesale*	41 (62.12)	48 (63.16)	89

*Retail*	25 (37.88)	28 (36.84)	53

**Time trading poultry**			

*Less than a year*	26 (39.39)	8 (10.53)	34

*Between 1-2 years*	6 (9.09)	14 (18.42)	20

*Between 2-3 years*	9 (13.64)	15 (19.74)	24

*More than 3 years*	22 (33.33)	39 (51.32)	61

*Missing/not responded*	3 (4.55)	0 (0)	

**Weekly frequency of poultry sale**			

*Every day*	14 (21.21)	21 (27.63)	35

*2-6 days per week*	48 (72.73)	55 (72.37)	103

*Once per week*	3 (4.55)	0 (0)	3

*Missing/not responded*	1 (1.52)	0 (0)	

**Type of poultry traded**			

*Chicken*	32 (48.48)	31 (40.79)	63

*Duck/muscovy*	7 (10.61)	10 (13.16)	17

*Mixed species*	15 (22.73)	22 (28.95)	37

*Missing/not responded*	12 (18.18)	13 (17.11)	

**Usually sells all poultry at market**			

*Yes*	56 (84.85)	41 (53.95)	97

*No*	9 (13.64)	35 (46.05)	44

*Missing/not responded*	1 (1.52)	0 (0)	

**Destinations of unsold poultry**			

*Other markets*	1 (11.11)	3 (8.57)	4

*Back to farms*	1 (11.11)	28 (80)	29

*Other traders*	0 (0)	3 (8.57)	3

*Directly to slaughter*	7 (77.78)	2 (5.71)	9

### Trade behaviour of LPT's and occurrence of HPAI outbreaks

A total of 43% (n = 56) communes with complete trade information (N = 131) had recorded HPAI outbreaks in poultry from 2003 through to 2006. There were only two positive communes in 2005-6 out of the 131 with disease data.

The results of the univariable and multivariable analysis of LPT attributes associated with HPAI outbreaks during 2003 to 2006 are presented in Tables 2 and 3 respectively. None of the tested biologically plausible first-order interactions resulted in improvement of model fit. The goodness-of-fit test showed a suboptimal fitness of the model (p = 0.177). The potential impact of clustering of data due to multiple measurements from each LPT resulted in an intra-cluster correlation coefficient of 0.547 (p < 0.001).

**Table 2:** Univariable results: commune infection status during 2003 to 2006 vs. LPT attributes.

Variable level	OR	SE	*P *value	95% CI	Overall *P *value
**Market type**					

*Wholesale*	Ref.				

*Retail*	15.541	12.03	<0.001	3.407, 70.883	

**Time trading poultry**					0.057

*Less than a year*	Ref.				

*1-2 years*	0.107	0.098	0.015	0.018, 0.646	

*2-3 years*	0.159	0.141	0.037	0.028, 0.898	

*>3 years*	0.232	0.171	0.047	0.055, 0.983	

**Direction of poultry trade**					

*Buy and sell*	Ref.				

*Sell only*	2.742	1.702	0.104	0.812, 9.258	

**Type of poultry traded**					0.461

*Mixed species*	Ref.				

*Chicken*	2.373	1.664	0.218	0.600, 9.378	

*Ducks*	1.776	1.475	0.489	0.349, 9.047	

*Muscovy ducks*	0.904	0.858	0.915	0.141, 5.808	

**Usual number of poultry traded**					0.008

*<60*	Ref.				

*60-90*	0.095	0.08	0.006	0.018, 0.501	

*90-150*	0.071	0.063	0.003	0.013, 0.400	

*>150*	0.099	0.083	0.006	0.019, 0.515	

**Flock size of contact farms**					0.621

*< 300*	Ref.				

*300-500*	0.598	0.351	0.381	0.189, 1.891	

*500-1,000*	0.488	0.269	0.193	0.165, 1.438	

*>1,000*	0.647	0.356	0.429	0.220, 1.902	

**Weekly frequency of poultry trade**					

*Every day of week*	Ref.				

*1 - 6 days per week*	0.132	0.103	0.009	0.029, 0.607	

**Usually sells all poultry at market**					

*Yes*	Ref.				

*No*	0.705	0.511	0.63	0.170, 2.923	

OR: Odds Ratio; SE: Standard Error; CI: Confidence Interval

**Table 3:** Multivariable results: commune infection status during 2003 to 2006 vs. LPT attributes.

Variable level	OR	SE	P value	95% CI	Overall *P *value
**Market type**					

*Wholesale*	Ref.				

*Retail*	14.99	10.624	<0.001	3.737, 60.129	

**Time trading poultry**					0.009

*Less than a year*	Ref.				

*1-2 years*	0.087	0.071	0.003	0.017, 0.434	

*2-3 years*	0.124	0.099	0.009	0.026, 0.595	

*>3 years*	0.186	0.123	0.011	0.051, 0.680	

OR: Odds Ratio; SE: Standard Error; CI: Confidence Interval

### Networks of poultry trade and occurrence of HPAI outbreaks Trader-commune network (Network 1)

**Figure 1:**
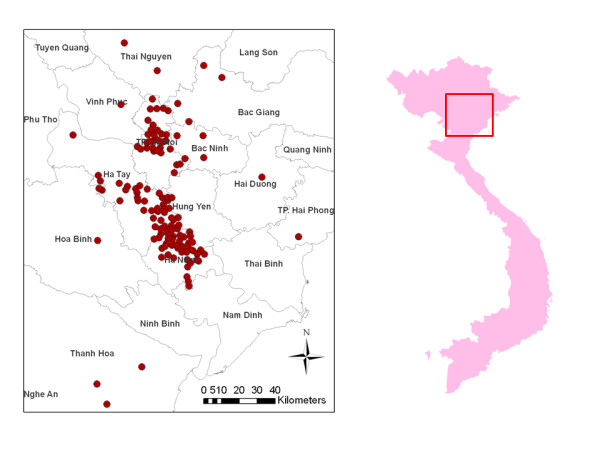
**Geographical distribution of the 131 communes of Vietnam contacted by 117 live poultry traders included in the social network analysis**. Inset: North Vietnam study area.

The geographical distribution of the communes included in the analysis of associations with commune infection status is shown in Figure 1. The 2-mode trader-commune network contains 308 nodes of which 191 are in the commune class and 117 in the LPT class, located in the following LBM's: Bac Thang Long, Cho Ni, Cho Phu Lo, Cho To, Da Ton, Ha Vi, Sui, and Yen Thuong. There were 474 flocks identified by LPT's in the set of communes; the average number of traded flocks is 2.48 per commune (average value of the links in commune class: 2.48, STD: 3.01, range: 1-29) and 4.05 per LPT (average value of the links in LPT class STD: 1.83, range: 1-10). There are 303 links between nodes and the density of the network is 2.12%. On average 1.58 traders operate per commune (average degree of the commune class; STD: 1.2, range: 1-10). LPT's trade on average in 2.59 communes (average degree of the trader class; STD: 1.3, range: 1-8). The network is very fragmented with 30 components, but there is a highly connected core of communes, consisting of a giant weak component and a sparse periphery (containing numerous isolated components of small size). The giant component includes 138 nodes (44.8%) linking 90 communes and 48 traders, the second component includes 43 nodes (14.0%), 21 communes and 22 traders and the other 28 components have 10 or less nodes with 19 components of five or less nodes.

### Commune-Commune networks (Network 2 and 3)

**Figure 2:**
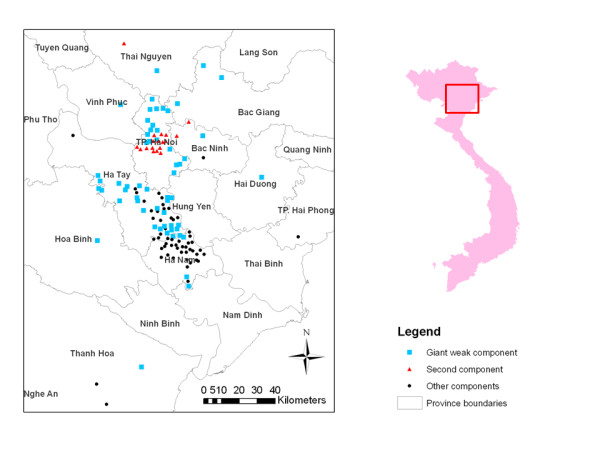
**Geographical distribution of the communes in the different components of the commune-commune network (Network 2)**. The highly connect core of communes (giant weak component) includes 44.8% (138/308) of nodes, linking 90(69%) communes and 48(41%) traders included in the study. The second component includes 14%(43/308) of nodes, linking 21(16%) communes and 22 (19%) traders included in the study; the other components (28 in total) have 10 or less nodes with 19 components of five or less nodes. Inset: North Vietnam study area.

This network is as equally fragmented as Network 1, consisting of 30 components; the locations of the communes in the two main components are shown in Figure 2.

The symmetric binary 1-mode commune network includes 191 nodes, has 1.77% density and 322 links. The average degree is 3.3 (STD: 2.3, range: 0-15) so that an average commune is connected to more than three other communes via common LPT's. The giant weak component includes 90 nodes (47.1%), and a second component has 21 nodes (11%). The remaining 28 components have 7 or less nodes with 25 components of five or less nodes. Network 2 contains 64 cliques: 34 3-cliques, 23 4-cliques, 5 5-cliques, 1 6-clique and 1 8-clique.

The clique overlap network (Network 3) contains the same number of communes, 191, with a density of 1.6% and 298 links. The average number of communes that share a clique with any other is 3.12 (mean degree: 3.12 STD: 2.53 Range: 0-15).

Applying significance tests to the subset of communes with attribute data, there was no significant difference in the mean degree of the nodes of Network 2 and 3 between the ones infected during at least one of the epidemic waves or in any single one (Table 4). The test of autocorrelation for Network 2 and 3 showed that the proportion of links between infected and non-infected communes (Type 2) is significantly lower than expected for the variable "Infected 2003-2006" (P = 0.039 and P = 0.06, respectively). The number of Type 1 and 3 links was not significantly different for the same variable (data not shown). Being a member of the giant component in Network 2 is significantly associated with not having been infected in any wave (χ^2 ^test: 14.14, 1 df, p < 0.001).

**Table 4:** Significance tests of the mean degree and number of Type 2 links for Networks 2 and 3 and different commune disease status.

Commune infection status	No	Yes	Two-tailed t-test probability of the difference of the mean degree		Difference between observed and expected Type 2 links and two-tailed test probability	
			**Network 2**	**Network 3**	**Network 2**	**Network 3**

**Infected 2003-6**	75	56	0.337	0.485	-18.16 (P = 0.039)	-16.16 (P = 0.06)

**Infected 2003-4**	79	52	0.308	0.404	-8.27 (P = 0.2)	-6.7 (P = 0.26)

**Infected 2004-5**	121	10	0.999	0.731	-10.12 (P = 0.07)	-8.726 (P = 0.123)

**Infected 2005-6**	129	2	0.439	0.387	-2.7 (P = 0.26)	-3.2 (P = 0.23)

### LBM-commune network (Network4)

**Figure 3:**
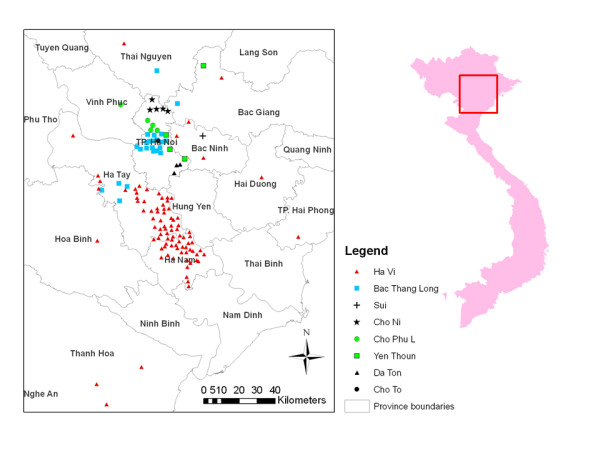
**Catchment area of the eight markets identified in Live Bird Market(LBM) - commune network (Network 4)**. The wholesale LBMs are Ha Vi and Bac Thang Long; the remainder are retail markets. Inset: North Vietnam study area.

The geographical location of the catchment areas of each LBM is presented in Figure 3. There are five communes linked to more than one LBM: two linked to Ha Vi and Bac Thang Long, one linked to Ha Vi and Cho Ni and two linked to Bac Than Long and Yen Thuong and Cho To, respectively. These five communes experienced AI outbreaks: three in 2003-4, one in 2004-5 and one in 2003-4 and 2004-5. This network consists of 199 nodes, representing links between 191 communes and 8 LBM's where the 117 LPT's of Network 1 were located.

In terms of trade volume, the two distinctive types of LBM, wholesale and retail, in Network 4 determine the network structure with one component linking the two wholesale LBM's and two retail LBM's as well as four isolated components corresponding to the catchment areas of the other four retail LBM's. In Ha Vi market, poultry from 315 flocks in 141 communes were traded mainly in the south of Ha Noi and scattered communes north of the capital, whereas in Bac Thang Long market poultry from 89 flocks in 31 communes were traded in a small area north of Ha Noi (Figure 3). The other six retail markets are local with small catchment areas in terms of number of flocks (range: 9-18) and communes (1-6).

## Discussion

To the best of our knowledge, this study represents the first investigation of the association between poultry trade and HPAI outbreaks in northern Vietnam. The results of this study highlight the advantage of combining a descriptive study with a network analysis of the characteristics of the poultry trade pattern of LPT's, and demonstrate that this methodology can improve our understanding of the epidemiology of HPAI H5N1 in affected countries. Although trade is usually difficult to quantify, the network analysis provided insight into the relational nature of the live poultry trade and its potential relationship with the spread and maintenance of AI in northern Vietnam.

This study supports previous research findings indicating that LBM's may constitute an important source of infection for poultry [18,20]. In particular, our analyses indicate that new LPT's (i.e. those trading for less than a year) and those operating in authorised retail LBM's have increased odds of sourcing poultry from flocks located in communes with past history of H5N1 outbreaks during 2003 to 2006, when compared to older LPT's (i.e. those trading for more than a year) and to those operating in wholesale markets. This suggests that individuals who are relatively new to the poultry trading business are more likely to operate in areas with previous infection history, perhaps due to their inexperience. It is also possible that experienced traders consciously avoid high disease risk areas, thereby providing new traders with an opportunity to set up their business in these areas. Although this finding does not allow inferences in relation to the source of the outbreaks amongst infected communes, it provides evidence for advocating modifiable practices directed towards new traders at retail markets. These could include the dissemination of historical and up-to-date information on the geographical distribution of outbreaks and the development of more biosecure poultry trading practices such as crate cleaning and disinfection.

Further, while it is a legal requirement that traders visiting an LBM for the purpose of selling poultry report to the market inspectors (MI) at the LBM's veterinary check point, those that leave the LBM with poultry are currently not required to report. As identified in our study, poultry are in some occasions introduced back to other flocks which present a risk for further virus transmission. We recommend that at the market-level, this type of poultry trade should be discouraged, or at least monitored by MIs. The implementation of a poultry traceability scheme would provide a mechanism for monitoring and regulating the traffic of live poultry in and out of the LBM's.

The links between specific communes in northern Vietnam were described using social network analysis. The LPT-commune network and the commune-commune networks showed low density of links and a typical core-periphery structure. While the presence of low density of links is beneficial from a disease control point of view, the presence of a highly connected core may pose considerable challenges for the geographical containment of disease when infected birds flow through these trade channels. Furthermore, we found that increasing numbers of LPT's operating in communes does not increase the risk of AI outbreaks during the period 2003-2006 nor does being linked to many other communes via the same LPT's. Although centrality measures at node level (such as the degree and membership of the giant component) have been suggested to be of practical use in the development of effective targeted disease control strategies [13,31], the investigation of the links within and between subgroups of nodes has provided better insight into the relationship between the disease status and network structure. The randomly permuted networks indicate that the observed number of links between communes of similar disease status is higher than the random distribution of links between communes, whereas the observed number of links between infected and non-infected communes is lower than expected. These results suggest that there is a separation in terms of the LPT's trading between infected and non-infected communes. Since in our network analysis the links between communes are defined by trading events of LPT's, these results indicate that the observed outbreak pattern in 2003-06 appeared to be associated to subgroups of LPT's, with communes linked by them having the same disease status. This finding was also supported by the analysis of the clique-overlap membership matrix.

The LBM-commune network showed that only a few communes (n = 5) traded poultry in both retail and wholesale markets. Nevertheless, all of these communes experienced AI outbreaks during 2003 to 2006, in contrast to 40.5% infection level amongst the other communes (n = 126). In addition, the travel distances from communes linked to retail markets are quite short compared to those linked to wholesale markets. This is consistent with the local emphasis of trade, where most of the LPT's in retail markets transport poultry in motorcycles fitted with baskets. Evidence from other livestock diseases suggests that fast long-range dissemination of infectious diseases can occur through live animal trade at large livestock markets [13]. Our descriptive analysis has shown that LPT's operating in wholesale markets tend to contact larger flocks and trade more poultry than those operating in retail markets. LPT's in wholesale markets generally travel by motorcycles with fitted crates, vans or lorries that enable larger distances and transport more animals than those operating in retail markets. Consequently, large wholesale markets can source and disseminate large quantities of poultry throughout a large area of northern Vietnam on the same day, further complicating disease control operations if infection were to flow through these channels. The dispersion of infection over a relatively large geographical space could be assisted by the trade with flocks in the few communes in the giant weak component that are geographically distant from its core.

Our results should be interpreted taking account the study's assumptions and limitations. Firstly, potential biases may have been introduced during the selection of LBM's and the LPT's. For example, by the time we carried out our surveys, only LBM's in the five outer districts of the capital city were allowed to trade poultry. Nevertheless, market selection was based on a history of recurrent HPAI H5N1 outbreaks, and on regional importance with respect to the magnitude of poultry production and trade within the Red River delta. In relation to the LPT's, the "buy only" was underrepresented because individuals leaving the LBM with live poultry do not have to report at the market veterinary check point. Secondly, while we conducted our survey during a period when poultry trade and therefore potential outbreak risk were both expected to be at their peak, the trade pattern may vary across seasons and years. Also, HPAI H5N1 disease control measures may change the trade pattern of live birds in and around the affected areas [28,32]. However, it is very difficult to deal with the uncertainty of the contact structure and its stability over time and through AI outbreaks. Even if the observed contact structure was different than prior to the AI outbreaks, it would be very difficult to ascertain whether this change was due to the impact of the disease, the control measure or pure changes in the trading behaviour caused by other reasons unaccounted for in this study (e.g. market value, changes in production). Only by simulating stochastically alternative network structures the impact of a wider range of outputs on the disease status could have been analyzed. However, there is not a network model that could represent a "standard" contact structure of live poultry trade in this area to which to compare the observed one. Thus, the poultry trade networks presented by this study are representative of the time period of peak poultry trade in North Vietnam and of the year when the study was conducted. Thirdly, our analyses are based on the assumption that the AI risk profile of a given LPT-flock link is associated with the historical infection status of the commune where the flock was located. This relationship is convenient because it enables the use of publicly available disease surveillance data aggregated at commune level. However, assuming that all flocks are a single population at risk could introduce systematic error to the interpretation of results. Factors such as commune area size and number of flocks in the communes may have an impact on the validity of this assumption by influencing the geographic dissemination of the virus within a commune. Currently in Vietnam, disease control policies based on flock depopulation consider village level depopulation as the main control measure for containing local virus dissemination, and thereby make the assumption that flocks in a village are a single population at risk. This decision represents a compromise between the logistic constraints of disease control and the known heterogeneity of poultry flocks among the approximately 16,000 communes of Vietnam. Moreover, analysis of population and area data of the communes included in this study, together with the known infectious properties of AI viruses, suggests that poultry flocks within communes may indeed be homogenous with respect to HPAIV H5N1 risk. Finally, the validity of a social network analysis based on our ego-centric data collection methods may have been influenced by sampling errors and lack of representativeness [33]. The statistical analysis could not include all nodes of the networks due to the lack of response of some LPT's and inability to correctly identify some communes named by them. The estimation of the standard errors and significance levels were affected by the fact that there were very few infected communes during the later AI epidemic wave (2005-06).

Taking these limitations into account, the results of this study indicate that the association of some LPT's to specific communes within the catchment area of authorised LBM's may support transmission of AI infection. This is particularly important where inadequate protection conferred by vaccination allows residual infection to remain in communes linked by LPT's of the trade network. These findings may support the need to promote policies encouraging more biosecure practices of LPT's operating through authorised LBM's. These could include a) the dissemination of information with respect to the geographic locations of high outbreak risk communes and b) the implementation of a formal data recording scheme for all incoming and outgoing LPT's. The former could be implemented by providing maps describing areas with previous history of outbreaks, and making them available, for example, at market veterinary checkpoints. Data recording systems should include regular data capture by the MIs concerning contextual characteristics of contact flocks (i.e. geographical location and type) and demographic characteristics of the trade (i.e. number of poultry traded and type). These interventions should be combined with enhanced flock-level biosecurity and disease monitoring and evaluation strategies to mitigate the risks associated with the modification of the LPTs' trade patterns. These interventions would have important implications for disease control efforts. At the local level, making disease outbreak information available to LPT's (particularly new traders and those at retail markets) would enhance their decision-making capacity when selecting geographical areas for trade. In addition, providing MIs with a data collection tool would formalise market access and contribute to their empowerment. These benefits would extend to the national-level by promoting enhanced knowledge of disease control operational managers regarding the live poultry trade structure in authorized LBM's.

## Conclusions

Our study provides evidence which is potentially important for informing policies intended to encourage more biosecure practices of LPT operating at authorised LBM's. Our study, which combined descriptive and network analyses, showed that:

• Less experienced traders (i.e. operating for less than a year) and those trading in retail markets are more likely to trade with areas with a history of HPAI H5N1 infection.

• Some LPT's introduce poultry to other flocks as part of their normal trade practice.

• Larger quantities of poultry are transported from a wider geographical area to wholesale markets when compared to retail markets.

• The association of some LPT's with a limited set of communes within the catchment area of authorised LBM's may support HPAI H5N1 transmission and may contribute to perpetuating HPAI H5N1 virus circulation among certain groups of communes.

Given the above, current disease prevention and control interventions would benefit from dissemination of information about outbreak risk and the implementation of a formal data recording scheme at LBM's for all incoming and outgoing LPT's.

## Methods

### Study population, data collection and dataset for analysis

In March 2007, a total of 12 live bird markets (LBM) were formally operating in greater Ha Noi: ten retail markets (Cho Ni, Cho Phu Lo, Cho To, Da Ton, Sui, Yen Thuong, Dong Ngac, Ngoc Hoi, Ngu Hiep and Tan Trieu) and two wholesale markets (Ha Vi and Bac Thang Long) that were located in the five outer districts of the capital city. A cross-sectional survey was carried out in all 12 markets between December 2006 and March 2007, which corresponds to peak poultry movement in Vietnam, due to high poultry demand around the traditional annual "Tet" festival holiday period. Based on informal interviews of market authorities conducted by the researchers and by officials of the Department of Animal Health of the Ministry of Agriculture and Rural Development, it was ascertained that a total of approximately 80,000 poultry are traded daily in these markets. This is estimated to represent approximately 75% of the total number of poultry traded during a day in the northern provinces of Vietnam.

The Market inspectors (MI) of the participating markets collected the data as they occupy a position of authority and accessibility to the LBM's and are the institutional link with the best knowledge of the local conditions of the LBM's. In Vietnam, official LBM's have at least one veterinary health worker who performs the activities of a MI. The MIs are employed by the District Department of Animal Health and posted at market veterinary checkpoints where they conduct and document animal health checks of poultry brought by incoming individuals. These individuals, by law, have to report to the market veterinary checkpoint prior to being allowed entry to the market. An interview was conducted by the MIs who had been previously trained in interview skills to enable a standardized data collection process. The interview was administered using a pre-tested structured questionnaire. The survey was conducted in each market for two days (i.e. a day prior to and on the day when poultry trade was considered more intense) in order to capture a representative sample of LPT's operating in each LBM. Based on a previous assessment of the trading pattern at each LBM it was noticed that despite LBM's usually operating daily, there is typically one day of the week when poultry trade is more intense [34]. During that day it is expected that most LPT's would be present. In that study it was also noticed that, with few exceptions, the same traders were operating in each market on a given day.

Data collection has followed an ego-centric approach whereby all trading activities of selected LPT's were collected. The survey elicited information from a total of 157 incoming and outgoing LPT's concerning (1) their poultry trade activities outside and inside the LBM and (2) their relationship with other intermediaries and poultry flocks. The questionnaire included questions regarding the usual number of poultry collected per flock in a day, the total number of poultry present at the flock(s), species of poultry reared in the flock(s) and the location of the flocks. We did not limit the number of flocks a LPT could name. The data on avian influenza outbreaks in Vietnam from 2003 through to 2006 was provided by the Epidemiology Unit of the Department of Animal Health, Ministry of Agriculture and Rural Development of Vietnam; the characteristics of this dataset are explained elsewhere [6].

### Statistical analyses

Based on previous knowledge of poultry husbandry practices at commune level and the available data from northern Vietnam on commune size and number of flocks per commune, the commune was considered an epidemiological compartment for disease status purposes [34]. For the statistical analysis of associations of LPT trade attributes with commune infection status during 2003 to 2006, and for the analysis of the poultry trade network we used the information generated from the "buy and sell" and "buy only" LPT's. This corresponded to a total of 142 LPT's (out of 157 initially interviewed) who had visited a total of 191 communes. Of the communes reported by LPT's it was possible to link the commune names to location data for 117(82%) LPT's.

These LPT's considered in the analyses were operating in eight LBM's (Bac Thang Long, Cho Ni, Cho Phu Lo, Cho To, Da Ton, Ha Vi, Sui, and Yen Thuong). The remaining four markets (Dong Ngac, Ngoc Hoi, Ngu Hiep and Tan Trieu) were visited by the 18 LPT's for which the location information was incomplete. From a total of 191 communes initially available we were able to ascertain AI outbreak status for the three waves for 131(68.5%) communes.

Associations between attributes of the LPT's were tested using the chi-squared test for trend when at least one of the attributes had more than two ordered categories. In addition, the association between attributes of the LPT's and the H5N1 infection status of communes where flocks were located was tested by fitting a random effect logistic regression model. The H5N1 infection status of the commune in the period 2003 to 2006 ("no outbreak" vs. "infection at least once") was considered as the outcome variable. The explanatory variables included "market type" ("wholesale" vs. "retail" markets), "time trading poultry", "direction of poultry trade" ("buy and sell" vs. "sell only"), "type of poultry traded", "usual number of poultry traded", "flock size of contact flocks", "frequency of poultry trade" and "usually sells all poultry at market". The effect of clustering due to multiple measurements at LPT's was assessed by including this variable as a random effect in the model. The statistical analysis was carried out in two phases using the LPT ID as the unit of analysis. Firstly, all LPT trade behaviour attributes were screened statistically using univariable logistic regression with commune infection status based on a p-value of 0.20, using the likelihood-ratio test. All continuous-scale variables were re-categorized into their quartiles. Secondly, all factors significant in the screening phase were considered for inclusion through a manual backward stepwise variable selection process using a multivariable logistic regression model. The criterion for removal of risk factors was based on the likelihood ratio statistic with a significance level of p > 0.05. The screening for the presence of confounding variables in the final model was performed by stepwise removal of variables which at some stage were not significant at p-value level of 0.05, and noting the impact on the coefficients of the remaining variables in the model. If one of the coefficients of the other variables had changed by more than 25% the eliminated variable was assumed to be a confounder and forced into the model [35]. Biologically meaningful first-order interaction terms were also tested for statistical significance. Model assumptions and goodness of fit were assessed following the methods described in Hosmer and Lemeshow (2000)[36]. All statistical analyses were performed using STATA 9.2 (Stata^® ^Corporation 2005).

Social network analysis was used to describe the connectivity pattern within the dataset consisting of records of paired trading events. Each pair represented the link between a particular LPT or LBM and the commune in which the source flock of the purchased poultry was located. Two symmetric 2-mode networks, valued and binary (Network 1), were built linking LPT's and communes so that two communes are linked via a LPT if they reported to have sourced from flocks in both communes during the study period. In this network nodes are divided into classes, LPT and communes, and in the case of the valued network includes the number of flocks in a commune the LPT traded with. The 2-mode trader-commune network was converted to a 1-mode binary symmetric network of communes linked via a common trader (Network 2). Basic descriptive measures of Network 1 and 2 were extracted: size, number of links, density and average non-normalized degree per class. The non-normalized degree measures the absolute number of unique links of a given node to the other node class. The components of the network were extracted and a binary variable was created identifying the communes included in the giant weak component (Yes/No). A component is a maximal connected sub-graph where all nodes (i.e. communes) are connected through paths [37]. Cliques of minimum size 3 were extracted and a symmetrised binary clique-overlap network (Network 3) was built where two communes are linked if they share at least one clique. A clique is a maximal complete sub-graph where each node is connected to all other nodes and the clique is not contained in any other clique [38]. This provided information about which nodes are more closely linked to one another than to other nodes of the network in tightly knit groups within the network [37,39]. Finally, two 2-mode symmetric networks, valued and binary (Network 4), were built linking communes and markets by replacing the mode 'trader' in Network 1 by the market where each LPT was identified and interviewed. Communes with degree greater than one were identified as a proxy for the identification of communes which are within the catchment area of more than one market.

To test the association of some network parameters and variables of commune infection status in three consecutive epidemic waves ("infection in 2003-4", "infection in 2004-5" and "infection in 2005-6" and "infection in any of the three waves"), several statistical tests adapted to network data were applied. The means of the degree distribution between infected and non-infected communes in the three outbreak waves were compared using the t-test with a permutation-based significance test involving 10,000 random permutations. The association between the density of the links in Network 2 and 3 and disease status of the commune was tested by applying a randomization test of autocorrelation for a symmetric adjacency matrix using two classes and 10,000 random permutations. The test of autocorrelation for Network 2 and 3 compared the observed number of links between two groups of nodes and the expected number obtained through random permutations of the network. The use of randomization tests of autocorrelation within symmetric adjacency matrices allows statistical significance testing of associations between dyadic binary variables such as represented by the links within the network and the disease status attributes of the commune nodes. The three types of links based on the different variables of disease status are: Type 1 - between infected communes, Type 2 - between infected and non-infected communes, and Type 3 - between non-infected communes. The association between disease status and membership in the giant component and the degree of commune in the Network 4 were tested using the Pearson's Chi-square test and the Fisher exact test (using the latter when the number of observations in any cell was less than 5).

All social network analyses were performed using UCINET 6.135 (^©^Analytic Technologies, Inc. 1999). Maps of the study area and the location of the communes included in the networks were produced using ArcView 9.2 (^©^ESRI).

## Authors' contributions

RJSM designed the study, conducted the analysis and drafted the manuscript. AO-P conducted the analysis and drafted the manuscript. DUP conceived the study, supervised the study and critically revised the manuscript. JO conceived the study and critically revised the manuscript. KLLT and QHD conducted the field work and performed data collection. All authors read and approved the final manuscript.
